# Treatment With 2-Pentadecyl-2-Oxazoline Restores Mild Traumatic Brain Injury-Induced Sensorial and Neuropsychiatric Dysfunctions

**DOI:** 10.3389/fphar.2020.00091

**Published:** 2020-02-25

**Authors:** Serena Boccella, Monica Iannotta, Claudia Cristiano, Fabio Arturo Iannotti, Fabio Del Bello, Francesca Guida, Carmela Belardo, Rosmara Infantino, Flavia Ricciardi, Mario Giannella, Antonio Calignano, Vincenzo Di Marzo, Sabatino Maione, Livio Luongo

**Affiliations:** ^1^ Department of Experimental Medicine, Pharmacology Division, University of Campania “L. Vanvitelli”, Naples, Italy; ^2^ Department of Pharmacy, School of Medicine, University of Naples Federico II, Naples, Italy; ^3^ Endocannabinoid Research Group, Institute of Biomolecular Chemistry, CNR, Pozzuoli, Italy; ^4^ School of Pharmacy, University of Camerino, Camerino, Italy

**Keywords:** traumatic brain injury, behaviour, electrophyiology, pain, plant metabolite

## Abstract

Traumatic brain injury (TBI) represents an important public health problem and is followed by neuroinflammation and neurological dysfunctions. It has been suggested that brain trauma is often associated to deep behavioral alterations and chronic pain-like syndrome. Despite inducing minimal brain damage, mild TBI (mTBI) leads to persistent behavioral changes, including anxiety, depression, social interaction impairment, and aggressiveness. The clinical management of these symptoms is still unsatisfactory and new pharmacological treatments are needed, especially for the aggressiveness and depression. In a mouse model of mTBI, we investigated the effect of 2-Pentadecyl-2-Oxazoline (PEA-OXA), a natural compound, that is a secondary metabolite, found in green and roasted coffee beans, on both the pain perception, and neuropsychiatric dysfunctions. We found that the compound acts as a α2 adrenergic antagonist and this mechanism is here described for the first time. Mild TBI mice, starting from 14-d post-trauma, developed anxious and aggressive behavior, whilst depressive-like behavior and impaired social interactions were observed from the 60th d onward. PEA-OXA normalized all the behavioral changes investigated. We also investigated the memory impairments through Morris Water Maze (MWM) test. Both sham and mTBI mice treated with PEA-OXA showed amelioration in the reversal task of the MWM. Nevertheless, the main symptom of the long-term mTBI is represented by the depressive-like behavior, which was completely reversed by PEA-OXA repeated administration. In humans, mTBI-induced depression precedes the appearance of dementias and is characterized by a massive deficit of GABAergic transmission in the cortices. We found that PEA-OXA normalized the GABA changes in the prefrontal cortex. In order to prove the α2-mediated effect of the PEA-OXA we have performed open field test in naïve animals by microinjecting into the medial prefrontal cortex the dexomedetomidine, a selective α2 agonist with or without PEA-OXA co-injection. We found that PEA-OXA antagonized the α2 agonist effect on the locomotor activity. Moreover, PEA-OXA microinjection into the medial prefrontal cortex induced an enhancement of dopamine release. Collectively, these data suggest that this natural compound, through its multi-target activity is able to: i) ameliorate behavioral alterations (i.e. depression), ii) selectively normalize cortical GABA levels, iii) rescue the impaired neuronal activity in the prefrontal cortex.

## Introduction

Traumatic brain injury (TBI) is an important public health problem. It may be associated to several neurological dysfunctions, inflammatory processes, and cell death (Arciniegas, 2011). Brain trauma is divided into two phases: an early injury and a secondary late reaction. For every 100,000 people in the population, about 500 people per year present to an emergency department with mild traumatic brain injury (mTBI)/concussion (Bazarian et al., 2005). Several animal models of TBI have been suggested (Shultz et al., 2016). It seems that neuro-inflammatory and pro-apoptotic processes occur in the early phase of mTBI (Zetterberg et al., 2013), whereas plastic phenomena, responsible for the change in neuronal activity, become evident in the late phase. The involvement of peripheral immune cells, such as mast cells and T-cells, has also been reported (Kelso and Gendelman, 2014; Corrigan et al., 2016). The secondary behaviors are associated with changes in brain activity, in particular in the medial prefrontal cortex (*m*PFC). The *m*PFC is also thought to play a key role in forebrain chronic pain-(Giordano et al., 2012; Luongo et al., 2013) and pain-associated behaviors (i.e., anxiety, depression, and cognitive impairments), which are often present as comorbidities of chronic degenerative diseases (Neugebauer et al., 2009). Although knowledge of the mechanisms involved in concussion is improving, the treatments are still unsatisfactory and new pharmacological tools are needed (Loane and Faden, 2010). We previously demonstrated that some of the central *sequelae* of mTBI could be treated, at least in part, with *N*-palmitoylethanolamide (PEA), a natural endogenous compound belonging to the fatty acid ethanolamide (FAE) family. However, in our hands PEA failed to counteract repetitive/anxious behaviours and had no effect on neuronal activity during the late phase of mTBI (Guida et al., 2017).

Several neurotransmitters and neuromodulators are involved in the pathophysiology of chronic conditions such as neuropathic pain (Yang and Chang, 2019), degenerative diseases (Kandimalla et al., 2018), and trauma (Guida et al., 2017). Recent evidence highlights a common soil for these pathologies, especially for the mechanisms responsible for the psychiatric component (Sandu et al., 2015). In the present study, we aimed to characterize the behavioral and electrophysiological phenotype of mTBI mice 60 days after injury, and to investigate the effect of a recently identified natural compound, i.e. 2-pentadecyl-2-oxazoline (PEA-OXA). This compound has been suggested to be anti-inflammatory in preclinical models of inflammation (Impellizzeri et al., 2016a; Petrosino et al., 2017) and to exert neuroprotective effect in an experimental model of Parkinson disease (Cordaro et al., 2018). Moreover, neuroprotective effects of PEA-OXA have been recently demonstrated in different animal models of ischemic brain damage (Impellizzeri et al., 2019; Fusco et al., 2019).

The mechanism through which PEA-OXA exerts its pharmacological effects is still poorly understood and seems to be different from that of its analogue, PEA. It has been suggested that PEA-OXA exerts an indirect effect on the endocannabinoid system as well as a neuroprotective effect through the modulation of the nuclear factor E2-related factor 2 pathways (Cordaro et al., 2018). Based on our previous study on mTBI (Guida et al., 2017), we have investigated the possible beneficial effects of chronic treatment with PEA-OXA on the behavioral, biochemical, and electrophysiological changes associated with concussion. Moreover, we also highlighted in this study a possible new mechanism of action of the compound in view of the possible involvement of neurotransmitters such as catecholamines. Therefore, even though the present study is based on the same experimental model and we performed several matched experimental procedures of our previous paper (Guida et al., 2017), here we tested a different compound, which is also a secondary plant metabolite of the green coffee, with a completely different pharmacodynamic profile that we identify for the first time.

## Materials and Methods

### Animals

Male C57BL/6J mice (Charles River, Italy) weighing 18–20 g were housed three per cage under controlled illumination (12 h light/dark cycle; light on 6:00 A.M.) and standard environmental conditions (ambient temperature 20–22° C, humidity 55%–60%) for at least 1 week before the commencement of experiments. Mice chow and tap water were available ad libitum. The Animal Ethics Committee of the University of Campania “L. Vanvitelli” and University of Naples “Federico II, Naples, approved the experimental procedures. Animal care was in compliance with Italian (D.L. 116/92) and European Commission (O.J. of E.C. L358/1 18/12/86) regulations on the protection of laboratory animals. All efforts were made to reduce both animal numbers and suffering during the experiments.

### Mild Traumatic Brain Injury (mTBI)

Experimental mTBI was performed using a weight-drop device developed in our laboratory. Mice were anesthetized with intraperitoneal injection of Tribromoethanol (250 mg/kg) and placed in a prone position on a spongy support. After a midline longitudinal incision, the skull was exposed to locate the area of impact and placed under a metal tube device where the opening was positioned directly over the animal's head. The injury was induced by dropping a cylindrical metal weight (50 g), through a vertical metal guide tube from a height of 20 cm. The point of impact was between the anterior coronal suture (bregma) and posterior coronal suture (lambda). Immediately following injury, the skin was closed with surgical wound clips and mice were placed back in their cages to allow for recovery from the anesthesia and mTBI. Sham mice were submitted to the same procedure as described for mTBI, but without release of the weight.

### Drugs

2-Pentadecyl-2-Oxazoline (PEA-OXA) and ultra-micronized (0.8–6.0 μm) Palmitoylethanolamide (PEA) were kindly provided by EPITECH Group SpA, Saccolongo (PD). PEA-OXA and PEA were dissolved in Kolliphor, used as vehicle, that was purchased by Sigma-Aldrich (Milan, Italy). PEA-OXA (10 mg/Kg i.p.), PEA or vehicle were systemically administered starting from the day after mTBI (day 1) induction until the end of experimental evaluations (day 60). For microdialysis experiments, a single injection of PEA-OXA or vehicle was orally or intraperitoneally performed at 10 and 20 mg/kg. For open field test, single intra-mPFC microinjections of ACSF, PEA-OXA or Dexmedetomidine (DEX) were performed at 6nmol/0.3 ul. Dexmedetomidine was purchased by Vetoquinol Italia S.r.l.

### Experimental Design

**Figure 1:**
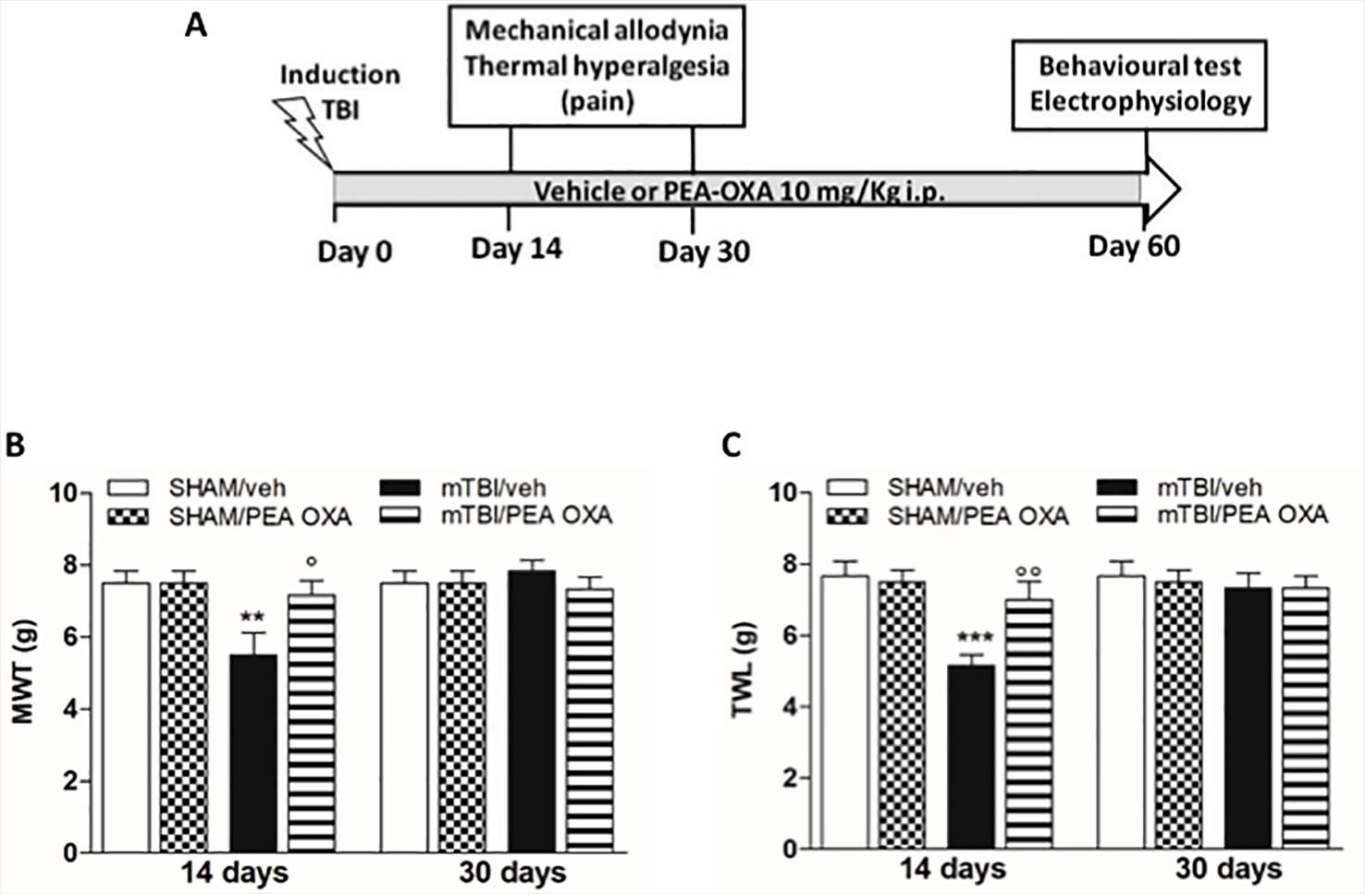
Effects of repeated administration of vehicle (Kolliphor 5%) or PEA-OXA (10 mg/kg, i.p.) on pain behavioral evaluations in sham and mTBI mice. **(A)** Timeline of the experimental procedure of mild Traumatic Brain Injury (mTBI) induction and related behavioral and electrophysiological characterization in presence of vehicle or PEA-OXA treatment. **(B)** Mechanical Withdrawal Thresholds (MWT) measured through Dynamic Plantar Aesthesiometer, **(C)** Thermal Withdrawal Latency (TWL) measured through the plantar test. Data are represented as mean ± SEM of 6–8 mice per group. **P < 0.01 and ***P < 0.001 indicate significant differences compared to SHAM/veh. °P < 0.05 and °°P < 0.01 indicate significant differences compared to mTBI/veh. *P*< 0.05 was considered statistically significant. One-way ANOVA, followed by Bonferroni *post hoc* test was performed.

Time points of evaluations were based on our previous study (Guida et al., 2017). A total number of 80 mice were divided in four experimental groups: SHAM/veh, mTBI/veh, SHAM/PEA-OXA, and mTBI/PEA-OXA. The day after mTBI induction, Behavioral tasks were performed at different time points and scheduled in order to avoid carry-over effects from prior testing experience. At the end of each set of experiments mice were sacrificed for further evaluations. The application of different pain stimuli -mechanical or thermal- was performed in separate groups of animals in order to avoid interferences in the nociceptive response. The timeline of mTBI induction, treatments, and behavioral and biochemical characterization is given in the **Figure 1A**.

### Mechanical Allodynia

Mechanical allodynia was evaluated at 14 and 30 days after mTBI or sham surgery by the Dynamic Plantar Aesthesiometer (Ugo Basile, Varese, Italy), as described by Guida et al., 2017. Mice were allowed to move freely in one of the two compartments of the enclosure positioned on the metal mesh surface. Mice were adapted to the testing environment for 30 min before any measurement was taken. After that, the mechanical stimulus was delivered to the plantar surface of the hindpaw of the mouse from below the floor of the test chamber by an automated testing device. A steel rod (2 mm) was pushed with electronical ascending force (0–30 g in 10 s). When the animal withdrawn its hindpaw, the mechanical stimulus was automatically withdrawn and the force recorded to the nearest 0.1 g. Nociceptive responses for mechanical sensitivity were expressed as mechanical withdrawal thresholds (MWT) in grams. Each mouse served as its own control, the responses being measured both before and after surgical procedures. An observer blind to the treatment quantified MWT.

### Thermal Hyperalgesia

Thermal hyperalgesia was evaluated at 14 and 30 days after mTBI or sham surgery by the Plantar Test Apparatus (Ugo Basile, Varese, Italy), as described by Guida et al., 2017. On the day of the experiment each mouse was placed in a plastic cage (22 cm × 17 cm × 14 cm; length × width × height) with a glass floor. After 30 min habituation period, the plantar surface of the hind paw was exposed to a beam of radiant heat through the glass floor. The radiant heat source consists of an infrared bulb (Osram halogen-bellaphot bulb; 8 V, 50 W). A photoelectric cell detected light reflected from the paw and turned off the lamp when paw movement interrupted the reflected light. Data were expressed as thermal withdrawal latency (TWL) in seconds and TWL was automatically displayed to the nearest 0.1 s; the cut-off time was 20 s in order to prevent tissue damage. Each mouse served as its own control, the responses being measured both before and after surgical procedures. An observer blind to the treatment quantified TWL.

### Marble Burying Test

Obsessive-compulsive behaviour was evaluated through the Marble burying test as previously described by D'Agostino et al., 2015. Mice were individually placed in a cage (42 cm × 24 cm × 12 cm length x width x height) containing 5 cm layer of sawdust bedding and 20 glass marbles (1.5 cm in diameter) arranged in four rows. Mice were left undisturbed for 15 min under dim light. An observer blind to the treatment counted the time spent in digging behaviour and the number of marbles buried (at least two or third buried in the sawdust). At the end of the test the animal was removed to its own cage.

### Tail Suspension Test

To assess the depression-like behaviour, tail suspension test was performed as previously described by Belardo et al., 2019. Mice were individually suspended 30 cm above the floor in a visually isolated area by adhesive tape placed approximately 4 cm from the tip of the tail. The duration of immobility, recorded in seconds, was recorded during the last 4 min of the 6-min test. Immobility time was defined as the absence of escape-oriented behaviour. Mice were considered to be immobile when they did not show any body movement, hung passively and completely motionless.

### Elevated Plus Maze Test

The elevated plus maze test has been performed as described by Marcinkiewcz et al., 2016. It consisted of two opened and two enclosed arms of the same size with 15-cm-high transparent walls. The arms and central square were made of white plastic plates and were elevated 55 cm above the floor. Arms of the same type were located opposite from each other. Each mouse was placed in the central square of the maze (5 × 5 cm), facing one of the closed arms. Mouse behavior was recorded during a 5-min test period. The number of entries into an arm and the time spent in the open and enclosed arms were recorded using a camera capture and analysed using a video tracking software (Any-maze, Stoelting, Wood Dale, IL, USA). The number of entries into open arms, the number of entries into closed arms and total distance travelled (m) were analysed.

### Light-Dark Box

Mice were tested to measure reckless behavior using light/dark box test, as described by Guida et al., 2017. The apparatus (60 cm × 30 cm × 30 cm; length × width × height) is divided into two equally sized compartments: the dark chamber was covered, whereas the light compartment was opened brightly illuminated (∼150 lux). Mice were placed in the light compartment and allowed to move freely between the two chambers for 10 min. The time spent in in each compartment, latency of first entry into the dark compartment and number of transitions were recorded.

### Resident-Intruder Test

At day 60 after mTBI or sham surgery, mice were tested for aggressive behavior using a resident intruder test as previously described by by Guida et al., 2017. Mice were individually housed for 1 week in Plexiglas cages to establish a home territory and to increase the aggression of the resident experimental mice. To begin, food containers were removed and an intruder mouse of the same gender was placed in a resident home cage and resident–intruder interactions were analyzed for 10 min. The aggressive behavior of resident socially-isolated mice was characterized by an initial pattern of exploratory activity around the intruder, which was followed by rearing and tail rattle, accompanied in a few seconds by wrestling and/or a violent biting attack. The analysis of aggressive behavior was performed by evaluating the following parameters: 1) number of attacks: each attack is characterized usually by the combination of at least two aggressive behaviors, including biting, chasing, wrestling, or lunging 2) anogenital sniffing: cumulative count for the test mouse to investigate the tail/anogenital area of the companion mouse. 3) social interaction: total count for the test mouse to approach the companion mouse, starting from a body separation distance of at least 1 cm apart (about half of the body width),

### Y-Maze Test

Spontaneous alternation is a measure of spatial working memory. Such short-term working memory was assessed by recording spontaneous alternation behavior during a single session in the Y-maze (made with three arms, 40 cm long, 120° C separate) positioned at exactly the same location for all procedures. Each mouse was placed at the end of one arm and allowed to move freely through the maze during a 5 min session. The series of arm entries were visually recorded. An arm choice was considered only when both fore paws and hind paws fully entered the arm. The Y-maze was cleaned after each test with 80% ethanol to minimize odor cues. Alternation was defined as a successive entrance onto the three different arms. The number of correct entrance sequences (e.g., ABC and BCA) was defined as the number of actual alternations. The number of total possible alternations was therefore the total number of arm entries minus two, and the percentage of alternation was calculated as actual alternations/total alternations × 100 (D'Agostino et al., 2012).

### Morris Water Maze

Morris water maze test has been performed as described by D'Agostino et al., 2015. It consists of a circular water tank (diameter 170 cm, height 60cm). The water temperature of 24 ± 1°C, light intensity and external visual cues in the room were rigorously reproduced. A circular clear Plexiglas escape platform (10 cm in diameter), was submerged 1.5 cm below the water surface. Swimming was recorded using a camera capture and analysed using a video tracking software (Any-maze, Stoelting, Wood Dale, IL, USA) that divided the pool into four equal quadrants: NE, SE, SW, and NW. The escape platform was placed in the midpoint of the SW and the position remained stable during 5 days. Mice were trained daily for four trials per day for 5 days, with inter-trial interval of 15 min and the start position was pseudorandomized across trials. Each trial terminated as soon as the animal reach the platform with a cut-off of 60 s. Average of the four trials for each mouse and then the average for each group to give a single path length and escape latency expressed as Mean ± SEM, was calculated for each training day.

A probe test was performed 1 h after the last swim on day 5. The platform was removed from the tank and each animal was allowed a free 60 s swim. The time spent in the quadrant where the platform was previously placed, was determined. A higher percentage of time spent in the platform quadrant is interpreted as a higher level of memory retention. Following the probe trial, reversal training was conducted for a further 3 days. During reversal training, the escape platform was moved to the midpoint of the opposite quadrant (NE) and, as for the training phase, mice were allowed to swim to this new position for four trials per day. A second probe trial was conducted 1 h after the last swim on day 8. All tests were conducted in the morning.

### Open Field Test (OFT)

Open field test has been set as described by Belardo et al., 2019. This test has been performed only in naïve mice in order to demonstrate a α2-mediated mechanism. The naϊve mice were implanted with a further cannula into the mPFC (AP: +1.42 mm; L: 0.5 mm; V: 3 mm) for microinjecting of Dexmedetomidine (Dex) or PEA-OXA 6 nmol/0.3μl. Behavioral assays were performed 10 min after drugs injection. The apparatus was cleaned before each behavioral session by solution of 70% EtOH. Naϊve mice were randomly assigned to a treatment group. Behaviors were recorded, stored, and analyzed using an automated behavioral tracking system (Smart v3.0, Panlab Harvard Apparatus). Mice were placed in an OFT arena (l × w × h: 25 cm × 25 cm), and ambulatory activity (total distance travelled in centimeter), were recorded for 20 min and analyzed.

### *In Vivo* Single Unit Extracellular Recordings

Single unit extracellular recordings have been performed as described by Guida et al., 2017. Mice for electrophysiological recordings were anaesthetized with Tribromoethanol (250 mg/kg) and placed in a stereotaxic device (David Kopf Instruments, Tujunga, CA). Body temperature was maintained at 37° C with a temperature-controlled heating pad. For electrode implantation, the scalp was surgically incised and the hole was drilled in the skull overlying the site of recording, *m*PFC (AP: +1.54–1.78 mm from bregma, L: 0.3–0.5 from midline and V: -1.5–3 mm below dura) according to the coordinates from the atlas of Franklin and Paxinos (1997). Anaesthesia was maintained with a constant continuous infusion of propofol (5–10 mg/kg/h, i.v.). A glass-insulated tungsten filament electrode (3–5 MX) (FHC Frederick Haer & Co., ME) was stereotaxically lowered into the prelimbic cortex (PLC) in *m*PFC. The recorded signals were amplified and displayed on a digital storage oscilloscope to ensure that the unit under study was unambiguously discriminated throughout the experiment. Signals were processed by an interface CED 1401 (Cambridge Electronic Design Ltd., UK) and analysed through Spike2 software (CED, version 4) to create peristimulus rate histograms (PSTHs) online and to store and analyse digital records of single-unit activity off-line. Configuration, shape, and height of the recorded action potentials were monitored and recorded continuously. This study only included neurons with a regular spiking pattern and a spontaneous firing rate between 0.1 and 3.82 Hz that were classified as pyramidal neurons in rodents (Jung et al., 1997; Tierney et al., 2004; Floresco and Tse, 2007). Once a neuron was single out, the position of the microelectrode was adjusted to maximize the spike amplitude relative to background noise and mechanical stimuli were applied. Mechanical stimuli were applied to the hind paw (contralateral to the *m*PFC) by Von Frey filaments with bending force of 97.8 mN (noxious stimulation) for 2 s (Simone et al., 2008). From the PSTHs, we measured neuron spontaneous activity measured in spikes/sec, the duration of excitation (in seconds) as the period of the increased firing activity, which exceeds the average baseline value +2SDs and the frequency of the evoked excitatory responses. Moreover, the extracellular action potentials' (EAP) amplitude, indicating synaptic current was used for the evaluation of the efficacy of synaptic transmission (Fagni et al., 1987).

### Microdialysis *In Vivo*

Microdialysis experiments were performed in awake and freely moving mice, as described by Belardo et al., 2019. In brief, mice were anaesthetized with Tribromoethanol (250 mg/kg) and stereotaxically implanted with concentric microdialysis probes into the *m*PFC using the coordinates: AP: +1.42 mm, L: 0.5 mm from bregma, and V: 3 mm below dura. Dialysis probes, were constructed with 25G (0.3 mm inner diameter, 0.5 mm outer diameter) stainless steel tubing (A-M Systems). Inlet and outlet cannulae (0.04 mm inner diameter, 0.14 mm outer diameter) consisted of fused silica tubing (Scientific Glass Engineering). The probe had a tubular dialysis membrane (Enka AG, Wuppertal, Germany) 1.3 mm in length. Following a recovery period of 24 h, dialysis was commenced with artificial cerebrospinal fluid (ACSF: KCl, 2.5; NaCl, 125; MgCl_2_, 1.18; CaCl_2_, 1.26, pH 7.2) perfused at a rate of 1 μl/min by a Harvard Apparatus infusion pump. Following a 60-min equilibration period, 12 consecutive 20–30 min dialysate samples were collected. After an initial 60 min equilibration period, dialysate samples were collected every 20–30min for 100–150 min to establish baseline release of L-glutamate (L-Glu), GABA and Dopamine (DA). At the end of the fifth dialysate sample, the drug (PEA-OXA 10-20 mg/kg or vehicle) was orally or intraperitoneally administered, and samples were collected for approximately 3 h. At the end of experiments, mice were anaesthetized and their brains perfused fixed *via* the left cardiac ventricle with heparinized paraformaldehyde saline (4%). Brains were dissected out and fixed in a 10% formaldehyde solution for 2 days. The brain was cut in 40-μm thick slices and observed under a light microscope to identify the probe locations. The concentrations of L-glutamate and GABA contained in the dialysate were analyzed using by HPLC coupled with fluorimetric detection method. The system comprised two Gilson pumps (model no.303), a C-18 reverse-phase column, and a Gilson fluorimetric detector (model no.121). Dialysates were pre-column derivatized with o-pthaldialdehyde-N-acetylcysteine (OPA-NAC) (10 μl dialysate + 5 μl OPA-NAC + 10 μl borate buffer 10%). The mobile phase consisted of two components: (A) 0.2 M Na2HPO4, 0.2 M citric acid and 20% methanol and (B) 90% acetonitrile. Gradient composition was determined using an Apple microcomputer installed with Gilson gradient management software. Mobile phase ﬂow rate was maintained at 1.2 ml/min. Data were collected using a Dell Corporation PC system 310 interfaced to the detector *via* a Drew data-collection unit.

Concentration of dopamine was determined using HPLC equipment fitted with an electrochemical detector. The composition of the mobile phase was 0.15 mM NaH_2_PO_4_, 0.01 mM octyl sodium sulfate, 0.5 mM EDTA (pH 3.8 adjusted with chloride acid), and 12.5% methanol. The mobile phase was delivered (flow rate: 1 ml/min) by a model 590 pump (Waters) into an Ultrasphere 5 μm ODS column (4.6 mm × 7.5 cm; Beckman Ltd). The electrochemical detector was an BIORAD mod. 1640, set at a potential of 0.55 V versus an Ag/AgCl reference electrode. The limit of detection for dopamine was 2–3 fmol per sample injected with a signal-to-noise ratio of 2. Data were expressed as mean ± SEM of 12 samples for each mouse of the fmol and pmol in 10 µl of perfusate.

### Cell Culture, Reagents, and Transfection

Fibroblast-like (COS-7) cells (ATCC CRL-1651) were grown in 24-mm plastic Petri dishes in Dulbecco's modified Eagle's medium (DMEM) containing 10% FBS, non-essential amino acids (0.1 mM), penicillin (50 U/ml), and streptomycin (100 µg/ml) in a humidified atmosphere at 95% O2/5% CO2 at 37°C. After plating, the cells were transfected on the next day with the plasmid encoding for the human adrenergic, α2B receptor (Origene Technologies, MD, USA; cat. RG220659) by use of Lipofectamine 2000 (Thermo Fisher, Milan, IT: cat. 11668027). A negative scramble control vector (Origene Technologies, MD, USA; cat. SKUGE100003) was used as control. The following day, the standard media was replaced with fresh DMEM containing G418 (Thermo Fisher, Milan, IT: cat. 10131027) to set up for the stable cell line selection. The sub-clones were generated using 0.6 ug/ml G418 in the media for one month. Sub-clones with the highest expression of the genes of interest were selected by quantitative PCR using the following primer sequences: a) human adrenergic alpha 2 receptor *forward:* GCCTCAACGACGAGACCTG *and reverse:* CCCAGCCCGTTTTCGGTAG and b) ribosomal protein S16 *forward:* TCGGACGCAAGAAGACAGC and reverse AGCAGCTTGTACTGTAGCGTG.

### RNA Purification and Quantitative Real-Time PCR

Total RNA was isolated from cells by use of the Pure Link RNA Mini Kit (Cat. N.: 12183018A; Thermo Fisher Scientific, Milan, Italy) following the manufacturer's instruction, and then quantified by spectrophotometric analysis. The purified mRNA was reverse-transcribed by use of iScript reverse transcriptase enzyme (Cat. N.: 1708840; Biorad, Milan, Italy). Quantitative real-time PCR was carried out in CFX384 real-time PCR detection system (Bio-Rad, Milan, Italy) with specific primers (see **Supplementary Table 1**) using Advance Universal SYBR Green Supermix (Cat. N.: 1725270 Bio-Rad, Milan, Italy). Samples were amplified simultaneously in quadruplicate in one-assay run with a non-template control blank for each primer pair to control for contamination or primer-dimers formation, and the ct (cycle threshold) value for each experimental group was determined. The housekeeping genes (the ribosomal protein S16) have been used as an internal control to normalize the ct values using the 2*^^−∆∆ct^* formula (Iannotti et al., 2010).

### Measurement of Cyclic AMP

Adenosine-3', 5'-cyclic monophosphate or cyclic AMP (cAMP) was measured in control and α2- COS transfected cells using to detect direct cyclic AMP immunoassay kit (Arbor Assays, Michigan, USA) following the manufacturer's instructions (Gosh et al., 2012).

### Statistical Analysis

Data were represented as mean ± SEM. Behavioral data were analyzed by using One-way or Two-way ANOVA, followed by Bonferroni's multiple comparison. Dunnet's *post hoc* test was used as *post hoc* test in microdialysis analysis. P values < 0.05 were considered statistically significant. Statistical analysis was carried out using Prism/Graphpad (GraphPad Software, Inc.) software.

## Results

### PEA-OXA Effects on Pain Behavior in mTBI Mice

A significant decrease of MWT and TWL was observed in vehicle-treated mTBI mice 14 days after trauma induction [MWT: 5.5 g ± 0.6, F(_7,40_) = 3.441, P = 0.0056; TWL: 5.2 s ± 0.3, F(_7,40_) =4.430, P = 0.0010] as compared to the sham group (MWT: 7.5 g ± 0.3; MWT; TWL: 7.7 s ± 0.4) (**Figures 1B, C**). No differences in pain threshold were observed between right and left paw (data not shown). Moreover, a complete physiological re-establishment of normal pain response was observed 30 days after trauma induction (MWT: 7.8 g ± 0.3; TWL: 7.3 s ± 0.4) as compared to the sham mice (MWT: 7.5 g ± 0.3; TWL: 7.7 s ± 0.4) (**Figures 1B, C**). Repeated administration of PEA-OXA (10 mg/kg, i.p.) significantly reduced both the mechanical allodynia and thermal hyperalgesia in mTBI mice 14 days after trauma (MWT: 7.2 g ± 0.4; TWL: 7.0 s ± 0.5) as compared to the vehicle treated mice (MWT: 5.5 g ± 0.6; TWL: 5.2 s ± 0.3) (**Figures 1B, C**), as revealed by One-way ANOVA followed by Bonferroni *post hoc* test. No difference was observed between right and left paw with PEA-OXA treatment (data not shown). PEA-OXA administration in sham mice did not change the pain response as compared to sham/vehicle mice at 14 and 30 days post trauma (MWT: 7.5 g ± 0.3; TWL: 7.5 s ± 0.3 and MWT: 7.5 g ± 0.3; TWL: 7.5 s ± 0.3, respectively).

### PEA-OXA Effects on Compulsive and Depressive-Like Behaviour in mTBI Mice

**Figure 2:**
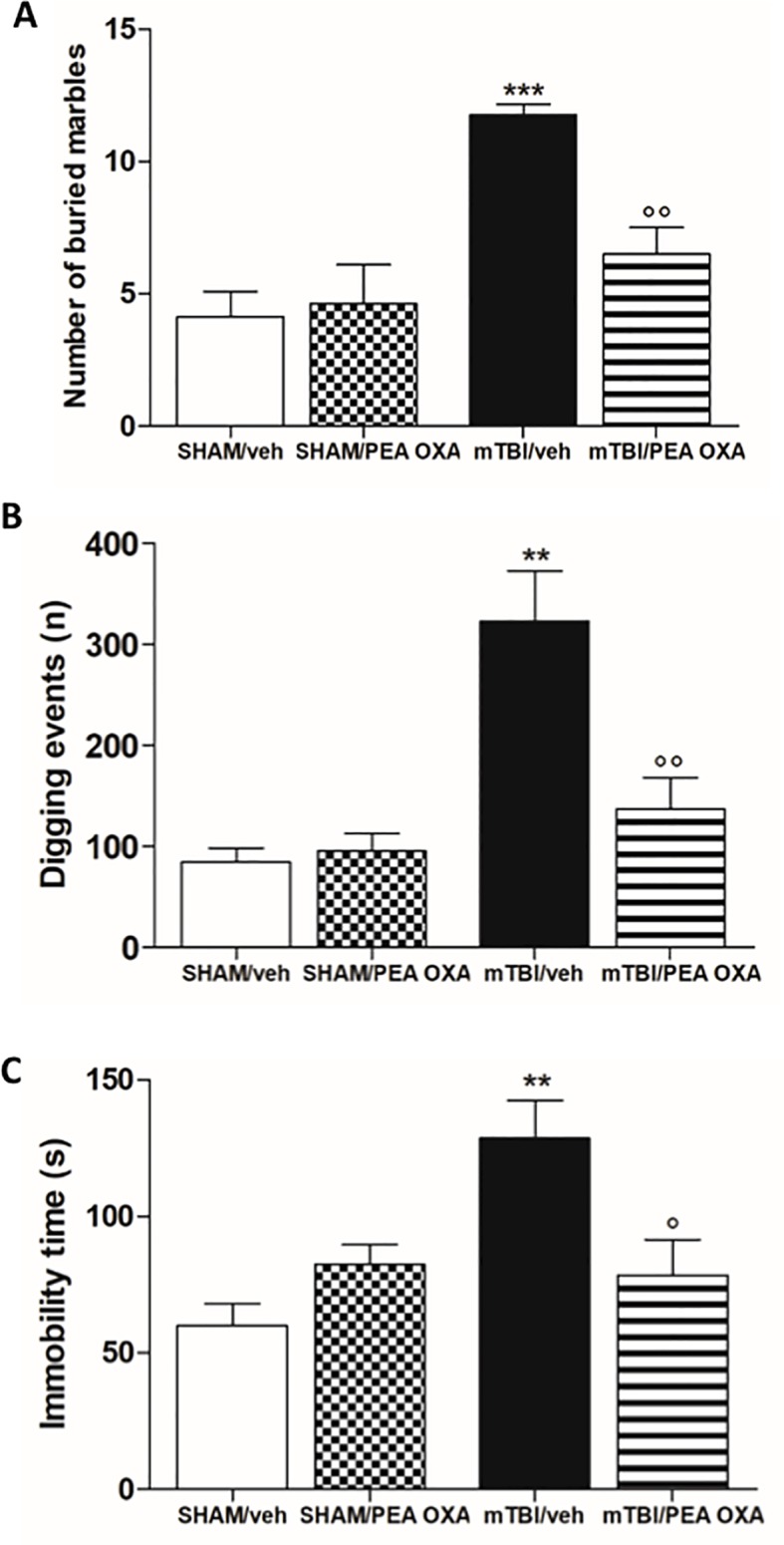
Effects of repeated administration of vehicle (Kolliphor 5%) or PEA-OXA (10 mg/kg, i.p.) on behavioral evaluations in sham and mTBI mice. **(A**, **B)** Number of buried marbles and digging events in marble burying test, respectively **(C)** Duration of immobility in the tail suspension test. Data are represented as mean ± SEM of eight mice per group. **P < 0.01 and ***P < 0.001 indicate significant differences compared to SHAM/veh. °P < 0.05 and °°P < 0.01 indicate significant differences compared to TBI/veh. *P*< 0.05 was considered statistically significant. One-way ANOVA, followed by Bonferroni *post hoc* test was performed.

At day 60 post trauma, mTBI/veh mice showed a significant increase of number of buried marble and digging events [11.8 ± 0.4 and 323.3 ± 50.0, F_(3,28)_ = 11.24, P = 0.0303 and F_(3,12)_ = 12.52, P = 0.0005 respectively], as compared to the sham/veh group (4.1 ± 0.9 and 84.5 ± 13.9 respectively) (**Figures 2A, B**). The treatment with PEA-OXA (10 mg/kg, i.p.) significantly reduced both parameters in mTBI mice (6.5 ± 1.0 and 137.3 ± 30.9 respectively) without inducing any change in sham mice (4.6 ± 1.5 and 95.5 ± 17.4 respectively), as revealed by One-way ANOVA followed by Bonferroni *post hoc* test.

Moreover, mTBI/veh mice showed an increased immobility time in the tail suspension test, measured as the lack of escape-oriented activity (128.5 ± 13.9 s) as compared to the sham/veh mice (60.0 ± 8.2 s) (**Figure 2C**). This depressive-like behaviour was significantly reduced by PEA-OXA treatment [10 mg/kg, i.p.; 78.5 s ± 13.1, F_(3,12)_ = 7.039, P= 0.0055] in mTBI mice, as revealed by One-way ANOVA followed by Bonferroni *post hoc* test. Sham mice treated with PEA-OXA did not show any change in immobility time as compared to vehicle treated mice (82.5 s ± 7.1) (**Figure 2C**).

### PEA-OXA Effects on Recklessness and Aggressive-Like Behavior in mTBI Mice

**Figure 3:**
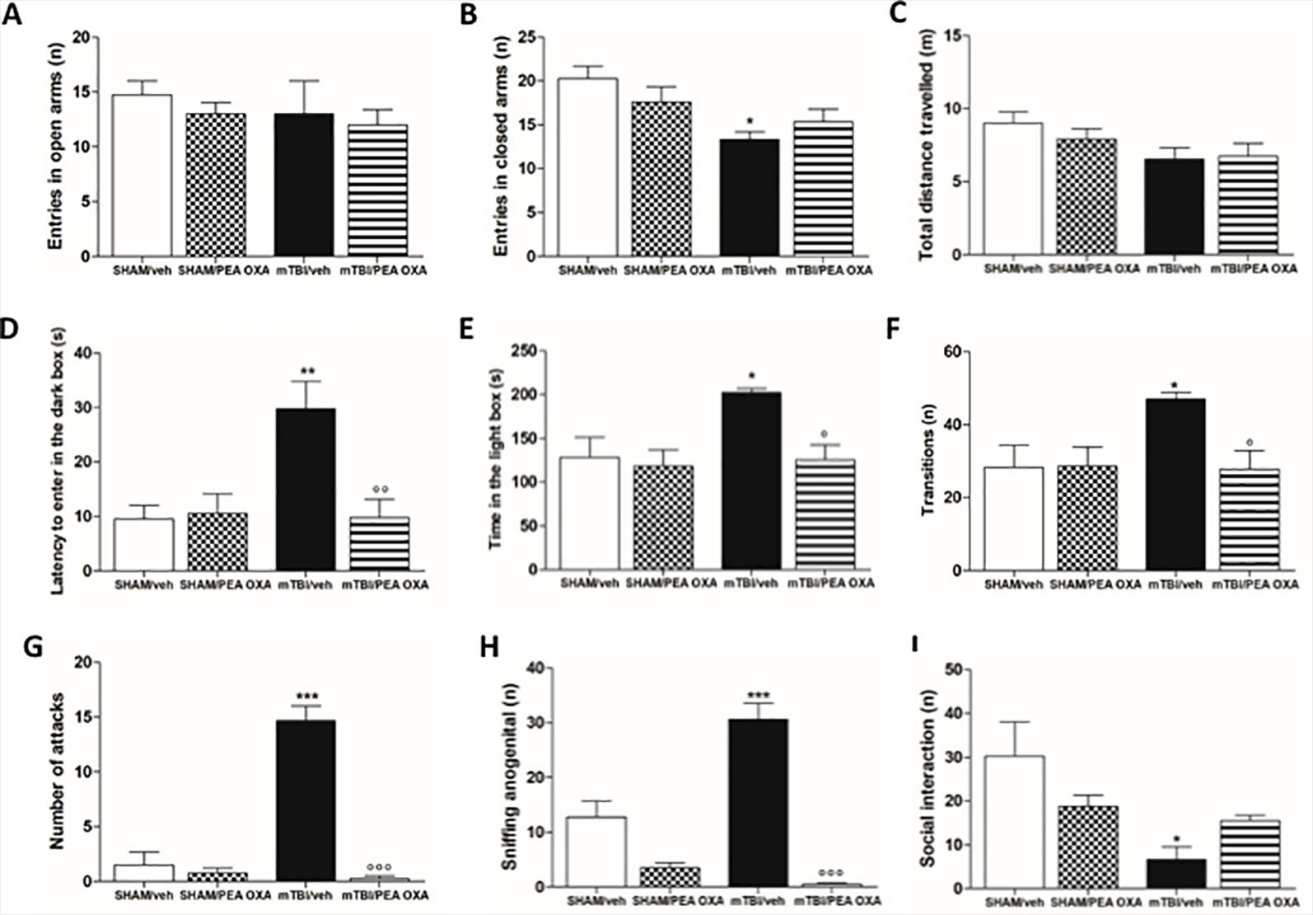
Effects of repeated administration of vehicle (Kolliphor 5%) or PEA-OXA (10 mg/kg, i.p.) on behavioral evaluations in sham and mTBI mice. **(A**–**C)** Entries in open arms, entries in closed arms and total distance travelled, respectively in elevated plus maze, **(D**–**F)** latency to enter in the dark box, time in the light box and transition, respectively, in light-dark box test, **(G**–**I)** number of attacks, sniffing ano-genital and social interaction, respectively, in resident intruder test. Data are represented as mean ± SEM of six to eight mice per group. *P < 0.05, **P < 0.01 and ***P < 0.001 indicate significant differences compared to SHAM/veh. °P < 0.05, °°P < 0.01, and °°°P < 0.001 indicate significant differences compared to TBI/veh. *P*< 0.05 was considered statistically significant. One-way ANOVA, followed by Bonferroni *post hoc* test was performed.

During the elevated plus maze test, although no significant differences were observed in the number of open arms entries (**Figure 3A**), mTBI/veh mice showed a significant lower number of entries in the closed arms (9.7 ± 0.3) as compared to sham/veh group (14.8 ± 1.2), indicating an anxiolytic-like effect [F_(3,14)_=3.021, P=0.0652] (**Figure 3B**). This effect was not reverted by PEA-OXA in mTBI mice (10 mg/kg, i.p.). Moreover, no differences between groups were observed in the total distance travelled (**Figure 3 C**). In the same way, in the light/dark box test, mTBI mice displayed a significant increase in the latency to enter in the dark box (29.8 s ± 4.9) as compared to sham/vehicle mice (9.5 s ± 2.5), which we identified as recklessness-like behaviour (**Figure 3D**). In this case, PEA-OXA did reduce the latency to enter in the dark box in mTBI mice [9.8 s ± 3.3; F_(3,14)_=6.963, P=0.0042]. Accordingly, mTBI/veh mice spent more time in the illuminated compartment of the light/dark box [196.8 s ± 7.6, F_(3,13)_ = 5.412, P = 0.0123] as compared to sham/veh mice (128.3 s ± 23.1), also showing an increased number of transitions in the two compartments [47.0 ± 1.8; F_(3,13)_ = 4.561, P = 0.0215] (**Figures 3E, F**) as compared to the control mice (28.2 ± 6.2). The treatment with PEA-OXA (10 mg/kg, i.p.) significantly decreased these effects (125.5 s ± 16.8, 27.7 ± 5.1) in mTBI mice (**Figure 3E, F**), as revealed by One-way ANOVA followed by Bonferroni *post hoc* test. Sham mice treated with PEA-OXA did not show any change in these paradigms as compared to the sham/veh mice (10.5 s ± 3.3; 118.3 s ± 18.7; and 28.5 s ± 5.4 respectively).

Moreover, mTBI/veh mice showed a significant increase in the number of attacks (14.7 ± 1.3) (1.5 ± 1.2) and ano-genital sniffing frequency (30.8 ± 2.9) during the resident intruder test, 60 days post trauma, as compared to the sham animals (**Figures 3G, H**). Additionally, they showed decreased number of social exploration (6.7 ± 2.9) as compared to the sham mice (30.2 ± 7.9) (**Figure 3I**). PEA-OXA administration (10 mg/kg, i.p.) reduced the number of attacks and ano-genital sniffing (0.3 ± 0.3, F_(3,11)_ = 54.40, P < 0.0001; 0.5 ± 0.3, F_(3,11)_ = 40.70, P < 0.0001, respectively) in mTBI mice, and increased the number of social interaction in mTBI mice, although One-way ANOVA followed by Bonferroni *post hoc* test analysis did not show a significant difference (15.5 ± 1.3) (**Figures 3G, H**). Finally, sham mice treated with PEA-OXA did not show any change in the number of attacks (3.5 ± 0.9), as compared to SHAM/veh mice (**Figure 3G**).

### PEA-OXA Effects on Spatial and Working Memory in mTBI Mice

**Figure 4:**
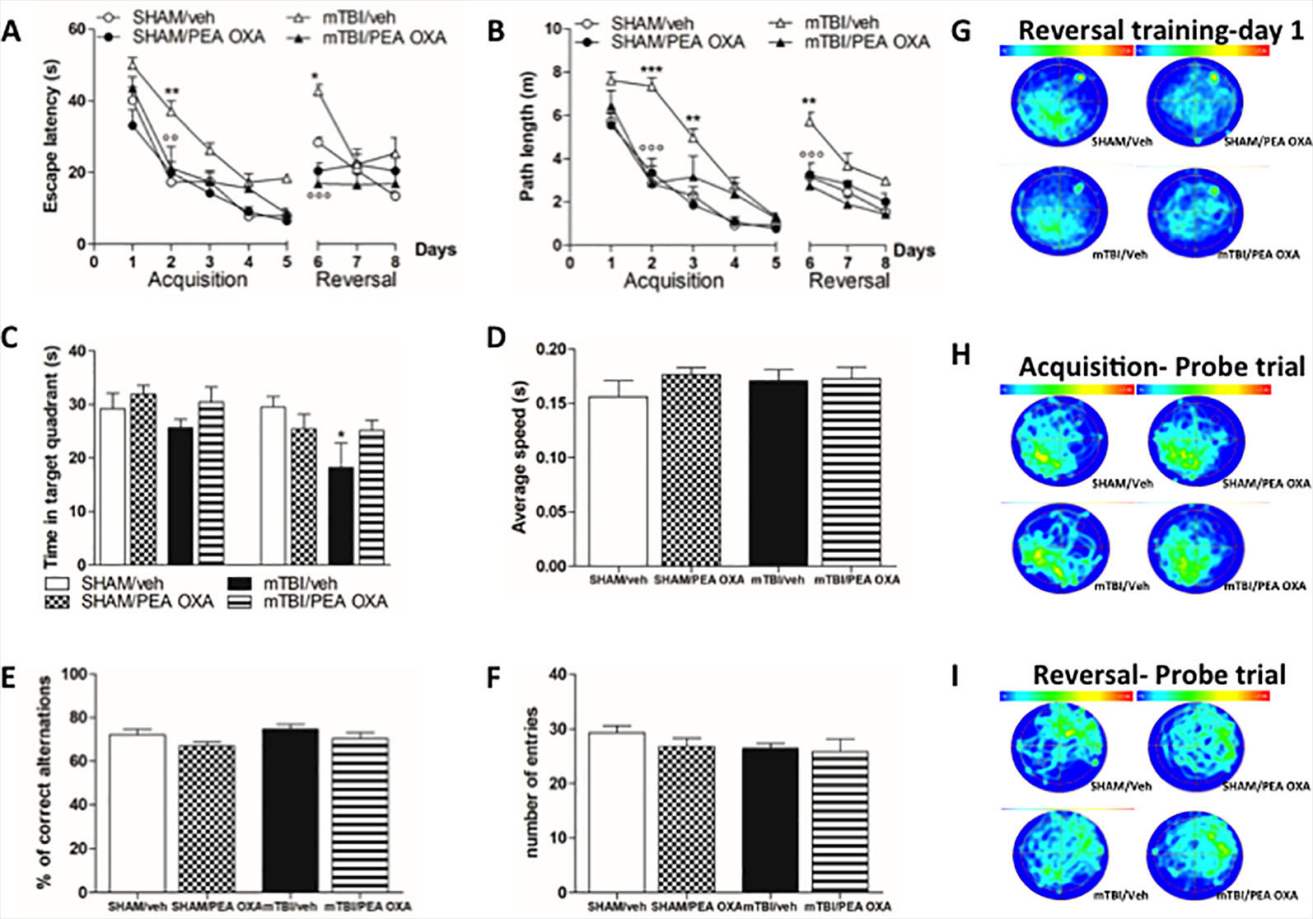
Effects of repeated administration of vehicle (Kolliphor 5%) or PEA-OXA (10 mg/kg, i.p.) on spatial and working memory in sham and mTBI mice. **(A**, **B)** Escape latency and path length, respectively, across 9 days of MWM reference platform task, **(C**, **D)** Time spent in the target quadrant and average speed, respectively, during probe tests of MWM test, **(E**, **F)** % of correct alternations and number of entries, respectively, in Y-maze test, **(G**–**I)** Occupancy plots during repeated MWM training and probe tests. Data are represented as mean ± SEM of eight mice per group. *P < 0.05, **P < 0.01, and ***P < 0.001 indicate significant differences compared to SHAM/veh. °P < 0.05, °°P < 0.01, and °°°P < 0.001 indicate significant differences compared to TBI/veh. *P*< 0.05 was considered statistically significant. One-way or Two-way ANOVA, followed by Bonferroni *post hoc* test was performed.

The effect of mTBI on spatial and working memory was examined by means of the Morris Water Maze and Y-maze tests. During the standard training trials, the escape latency (**Figure 4A**) and path length (**Figure 4B**) were longer in mTBI/veh mice especially on day 2 (37.1 s ± 2.9 and 7.3 ± 0.4, respectively) than that observed in sham/veh mice (17.3 s ± 0.7 and 2.8 s ± 0.1, respectively), and on day 3 in path length (4.9 s ± 0.4) as compared to sham/veh mice (2.3 s ± 0.4).

Conversely, mTBI mice treated with PEA OXA (10 mg/kg, i.p.) showed a significant decrease in the escape latency in mTBI/veh mice on day 2 (21.2 s ± 6.0, P < 0.01 and 2.8 ± 0.8, P < 0.001, respectively). Two-way ANOVA followed by Bonferroni post-hoc test revealed significant differences for time in both parameters [F_(7,105)_ = 45.91, P < 0.0001 and F_(7,105)_ = 53.93, P < 0.0001, respectively], for treatment x time interaction [F_(10,105)_ = 2.30, P = 0.003], but no significant differences were for treatments [F_(3,105)_ = 7.51, P = 0.0027 and F_(3,105)_ = 11.63, P = 0.0003, respectively] (**Figures 4A, B**). On day 6, we moved the platform in the opposite quadrant (NE) to analyse the time that mice spent in learning the new position. During the first day, sham (28.4s ± 1.4 and 3.2 ± 0.2, respectively) and mTBI mice treated with PEA-OXA (16.9 s ± 3.8 and 2.7 ± 0.3, respectively), were able to adapt their spatial strategies to find the new platform position very quickly, whereas mTBI/vehicle mice (42.7 s ± 1.8 and 5.7 ± 0.4, respectively) largely failed to deploy spatial strategies during this initial phase of reversal learning. After 3 days of training the mTBI/veh mice (25.3 s ± 4.4 and 2.9 ± 0.2, respectively) still spent more time to get the platform position as compared to sham group (13.4s ± 1.3 and 1.5 ± 0.4, respectively) even though the difference was not statistically significant. PEA-OXA administration (10 mg/kg, i.p.) in sham mice did not reveal any change in both phases (**Figures 4A, B, G**).

Probe trials were held to assess spatial reference memory during the acquisition and reversal phases and the percentage of time spent in the correct quadrant was measured (**Figure 4C**). Although there was a downward trend in the percent of time spent in the correct quadrant for the mTBI/veh mice (25.7 s ± 1.5) as compared to sham/vehicle mice (29.2 s ± 2.9), Bonferroni post-hoc test did not reveal significant differences between groups after the acquisition phase. Conversely, the probe after the reversal phase revealed a significant deficit in mTBI/veh [18.2 ± 4.6, F_(7,29)_=2.477, P=0.0402] as compared to sham/veh mice (29.5 s ± 1.9), which was partially restored by PEA-OXA treatment in mTBI mice (25.2 s ± 1.9, t = 1.855). However, heat maps of probes conducted after acquisition (**Figure 4H**) and reversal (**Figure 4I**) trials illustrated that all groups of mice finally developed a focal search pattern, although sham and treated mice rapidly focused on the target quadrant, whereas mTBI mice displayed a more diffuse and confuse search pattern. Notably, swimming speed was not different between the groups indicating that mTBI did not affect swimming ability [F_(3,16)_= 0.5678, P = 0.6442] (**Figure 4D**).

Finally, to determine whether mTBI led to working memory impairment, we performed Y-maze test. There were no differences in % of correct alternations during the spontaneous alternation in the Y-maze test [F _(3,16)_ = 1.984, P = 0.1571] (**Figure 4E**) and in the number of arms entries [F _(3,16)_ = 0.9421, P = 0.4435] (**Figure 4F**).

### PEA-OXA Effect on *m*PFC(+) Neurons in mTBI Mice

**Figure 5:**
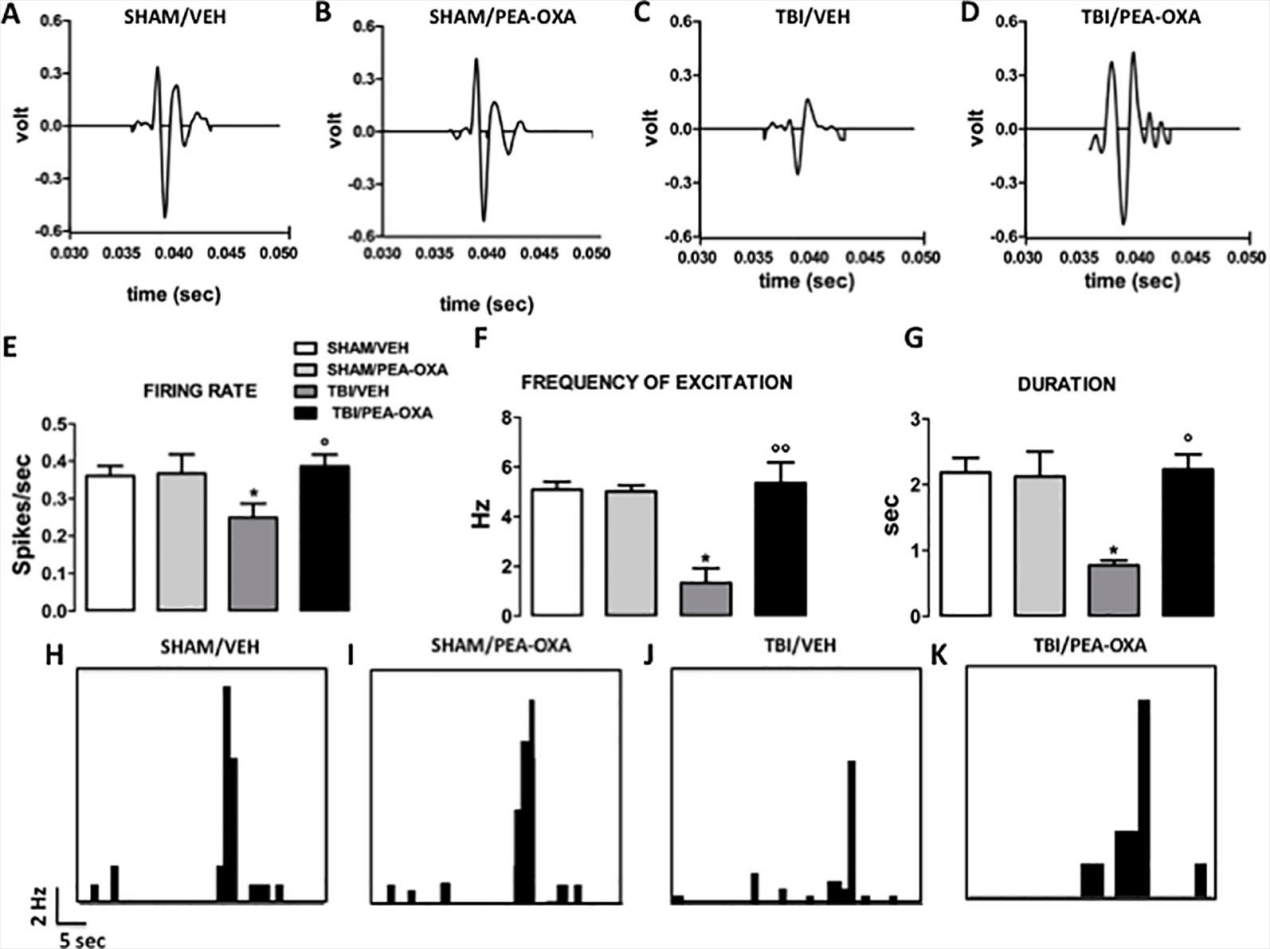
Effects of repeated administration of vehicle (Kolliphor 5%) or PEA-OXA (10 mg/kg, i.p.) on spontaneous and mechanical stimulus-evoked excitation of single *m*PFC (+) neuron in SHAM and TBI mice 60 days after trauma **(A**–**D)** Representative action potential of single *m*PFC (+) neuron SHAM and TBI mice treated with vehicle or with PEA-OXA, **(E**–**G)** Mean of spontaneous activity, of the frequency and of the duration of excitation, respectively, in different groups of mice, **(H**–**K)**. Representative PSTHs of *m*PFC (+) neuron of SHAM and TBI mice 60 days post trauma treated for 60 days with vehicle or PEA-OXA. Data are represented as mean ± SEM of six to eight mice per group. *P < 0.05 indicate significant differences compared to SHAM/veh. °P < 0.05 and °°P < 0.01 indicate significant differences compared to TBI/veh. *P*< 0.05 was considered statistically. One-way ANOVA, followed by Bonferroni *post hoc* test was performed.

As previously demonstrated (Guida et al., 2017), TBI mice, 60 days after induction of trauma (TBI/VEH), showed an important *m*PFC hypoactivation (**Figure 5**). In fact, a significant reduction of frequency [1.32 Hz ± 0.59, F _(3,21)_ = 5.87, P = 0.0045] and of duration of evoked activity (0.77 s ± 0.07, F_(3,21)_ = 3.09, P = 0.037) was observed, as revealed by One-way ANOVA followed by Bonferroni *post hoc* test (**Figures 5F, G**). Also, the spontaneous activity displayed a trend to reduction as compared to sham/Veh group (0.25 spikes/sec ± 0.03, F_(3,21)_ = 1.88, P = 0.17) (**Figure 5E**). Repeated treatments with PEA-OXA (10 mg/kg, i.p.) did not affect either spontaneous (0.36 spikes/sec ± 0.05) or stimulus-evoked activity (5.0 Hz ± 0.24 for frequency and 2.12 s ± 0.37 for duration) of *m*PFC (+) neurons of sham mice (**Figures 5E–G**). Instead, treatment with PEA-OXA (10 mg/Kg, i.p.) normalized the frequency (5.35 Hz ± 0.82) and the duration of excitation (2.23 s ± 0.22) in *m*PFC (+) neurons of mTBI mice. Finally, the same treatment increased spontaneous activity rate (0.38 spikes/sec ± 0.03) as compared to mTBI/veh mice (**Figures 5E, J, K**). These effects are also represented in PSTHs samples of single *m*PFC (+) neurons in SHAM and mTBI mice 60 days post trauma treated for 60 days with vehicle or PEA-OXA (**Figures 5H–K**).

### PEA-OXA Effect on Amino Acid Release in *m*PFC of Naϊve Mice

**Figure 6:**
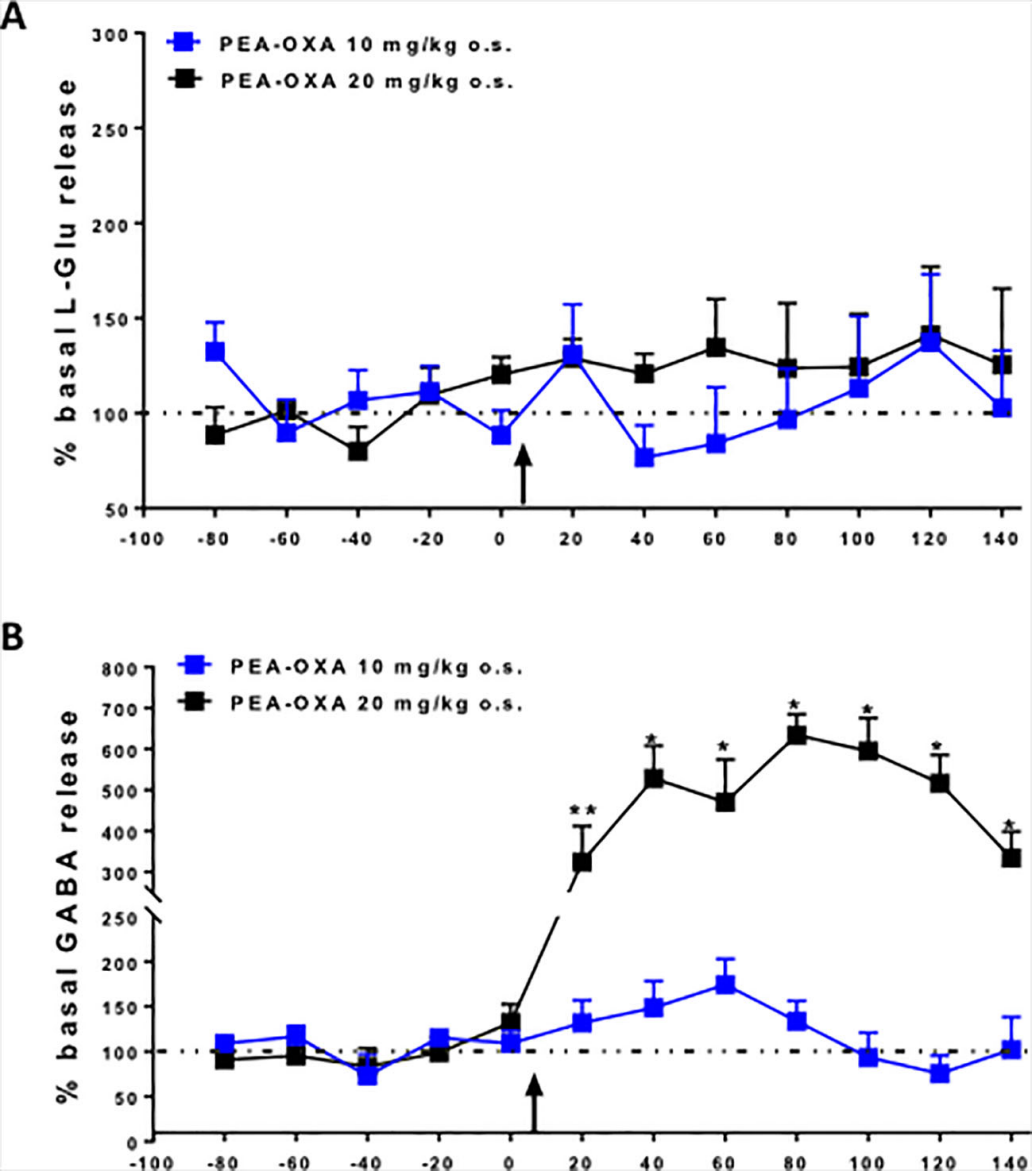
Effects of single injection of PEA-OXA (10 and 20 mg/kg, o.s.) on L-glutamate **(A)** and GABA **(B)** extracellular levels in *m*PFC of Naϊve mice. Each point represents the mean ± S.E.M of 3-4 animals per group. *P < 0.05 and **P < 0.01 indicate significant differences versus time 0. The black arrow indicates the administration of the drugs. P values < 0.05 were considered statistically significant. One-Way ANOVA, followed by Dunnett's *post hoc* test for multiple comparisons test was performed.

The effects of single injection of PEA-OXA (10 or 20 mg/kg *o.s.*) on L-Glu and GABA extracellular concentrations in the *m*PFC are shown in **Figures 6A, B**. Baseline levels of L-Glu and GABA were 7.0 pmol/10μl ± 0.6 and 2.3 pmol/10μl ±0.2 (data not show), respectively, in the *m*PFC of naϊve mice. We found that acute administration of PEA-OXA, at either dose, did not significantly change L-Glu extracellular levels in the *mPFC* of Naϊve mice [F_(7,16)_=0.6018, p=0.7560 for 10 mg/kg; F_(2,7)_=0.6934, p=0.4981 for 20 mg/kg] (**Figure 6A**). On the other hand, when PEA-OXA was administered at the highest dose (20 mg/kg), GABA levels significant increased in the *m*PFC (time 80 min: 508.4% ± 107.4 *vs* time 0: 100% ± 4.4, p=0,0036, F_(3,7)_=14.52) as revealed by One-way ANOVA followed by Dunnett's *post hoc* test for multiple comparisons within groups. Instead, extracellular levels of GABA in the *m*PFC did not significantly change after a single injection of PEA-OXA at 10 mg/kg o.s. [F_(7,16)_=1.487, p=0.2409] (**Figure 6B**).

### *In Vitro* Results

**Figure 7:**
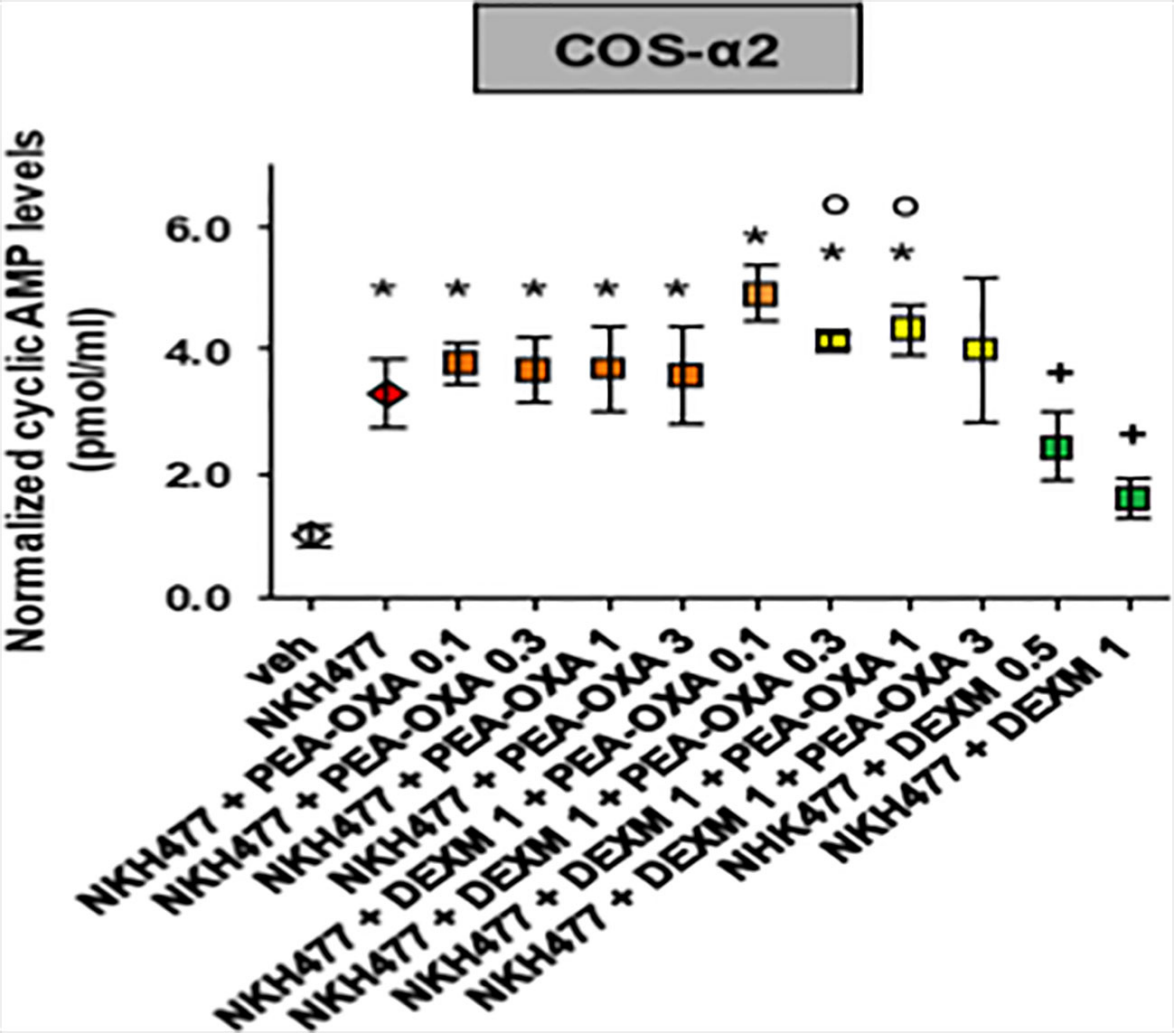
Effect of PEA-OXA in COS cells stably expressing human adrenergic α2 receptors. Scatter plots showing the effect of PEA-OXA in COS cells expressing α2 receptors on intracellular AMPc levels. Data represent the mean ± S.E.M. of four/five separate determinations. Data sets were compared using one-way ANOVA followed by Bonferonni's test. *** p value ≤ 0.001 vs the indicated experimental groups; *p value ≤ 0.05 vs vehicle; **^+^**p value ≤ 0.05 vs NKH+PEA-OXA; **°**p value ≤ 0.05 vs NKH+DEXM.

To investigate for the potential regulation of adrenergic (α2B) receptors by PEA-OXA, we generated human α2B stably transfected COS cells. Next, we measured potential changes in the cyclic adenosine-3', 5'-cyclic monophosphate or cyclic AMP (cAMP), being these subclasses of receptors coupled to G_i_ protein dependent mechanisms. By means of an immune-enzymatic assay, we found that PEA-OXA (from 0.1 to 3 µm) did not interfere with the elevation of cAMP induced by NKH477, a water-soluble enhancer of cAMP. However, PEA-OXA (from 0.1 to 3 µM) counteracted the inhibitory effect on cyclic AMP of dexmedetomidine, a selective α2 agonist, suggesting that PEA-OXA could be considered as a novel antagonist of α2 adrenergic receptors (**Figure 7**). This was also confirmed by the results of binding assays with PEA-OXA for α2 adrenergic receptors (See **Supplementary Materials**).

### Single Administration of PEA-OXA in mPFC Ameliorated Low Motor Activity Deficits in Open-Field Exploration Induced by α_2_-Agonist

**Figure 8:**
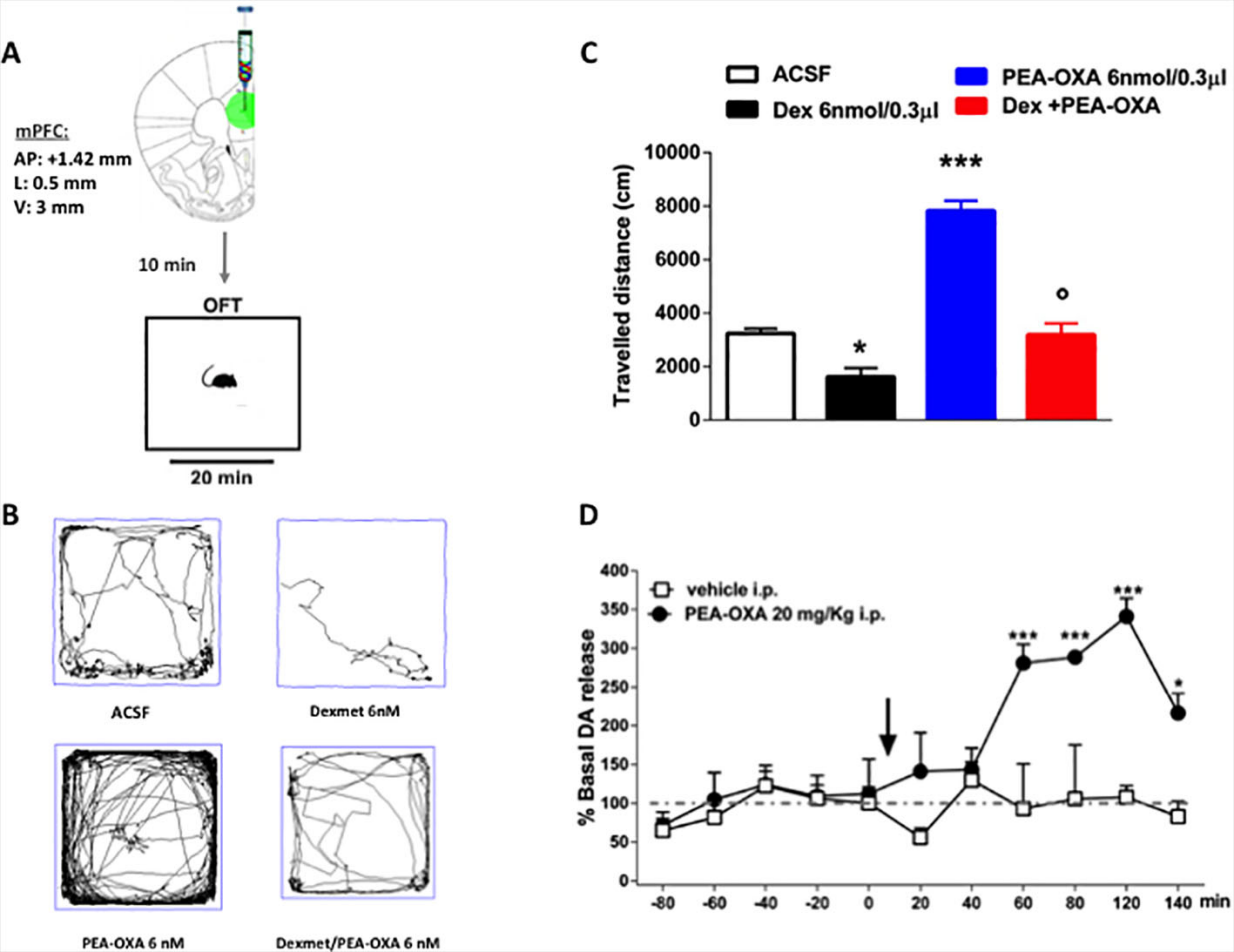
Effects of single injection of PEA-OXA (6 nmol/0.3 μl i.c.v. and 20 mg/kg i.p.) on dexomedetomidine-induced decreased locomotor activity and on Dopamine extracellular levels in *m*PFC of Naϊve mice. **(A)** Representation of coronal sections of the mouse brain with the cannula placement in mPFC, **(B)** Representative traces of mouse movement during an open field test, **(C)** Total distance traveled in OFT. Data are represented as mean ± SEM of 3 mice per group. *P < 0.05 and ***P < 0.001 indicate significant differences compared to ACSF. P < 0.05 indicate significant differences compared to PEA-OXA 6 nM. *P* < 0.05 was considered statistically. One-Way ANOVA, followed by Tukey's *post hoc* test for multiple comparisons test was performed. **(D)** Dopamine extracellular levels in mPFC of Naϊve mice. Each point represents the mean ± S.E.M of four animals per group. *P < 0.05 and ***P < 0.001 indicate significant differences versus time 0. °P < 0.05 indicates significant differences compared to PEA-OXA 6 nmol/0.3 μl. *P*< 0.05 was considered statistically. Two-Way ANOVA, followed by Bonferroni's *post hoc* test for multiple comparisons test was performed.

In order to confirm the *in vitro* results and the binding assay we performed the open field task in freely moving naïve mice by microinjecting into the medial prefrontal cortex (mPFC) the selective α2-agonist dexmedetomidine alone or in combination with PEA-OXA (**Figure 8A**). In the open field test, Naϊve mice microinjected with Dex 6nmol/0.3μl in mPFC showed a significant decrease in the travelled distance (1618 ± 330.7 cm, one way-ANOVA followed by Tukey's post-hoc test) compared with Naϊve mice treated with ACSF [3237 ± 181.3 cm, p < 0.0001, F_(3,8)_ = 62.24].

By contrast, Naϊve mice that received PEA-OXA 6nmol/0.3μl microinjection in mPFC showed a significant increase in the ambulatory activity (7827 ± 375 cm, one way-ANOVA followed by Tukey's post-hoc test) compared with Naϊve mice that received ACSF [3237 ± 181.3 cm, p < 0.0001, F_(3,8)_=62.24]. The co-injection of Dex and PEA-OXA, at the same concentration, produced a normalization in the travelled distance (3186 ± 423.2 cm, one way-ANOVA followed by Tukey's post-hoc test) compared with Naϊve mice that received Dexmedetomidine alone [1618 ± 330.7 cm, p < 0.0001, F_(3,8)_ = 62.24], confirming the α_2-_mediated effect of PEA-OXA (**Figures 8B, C**).

### Effect of Single Administration of PEA-OXA on Dopamine Release in the mPFC of Naϊve Mice

To evaluate a possible neurochemical substrate associated with the increased locomotor activity induced by PEA-OXA, we have measured dopamine levels after PEA-OXA cortical microinjection, through microdialysis *in vivo*. The effects of single injection of PEA-OXA (20 mg/kg i.p.) on dopamine extracellular concentrations in the mPFC are shown in **Figure 8D**. Baseline levels of dopamine 8.92 pmol/10μl ± 0.58 in the mPFC of naϊve mice. Two-way ANOVA revealed that the single injection of PEA-OXA significantly increased the dopamine levels in the mPFC, from 60 min until 140 min, in naïve mice compared to the vehicle treated mice [time 120: 340.87 ± 23.22% vs 108.20 ± 15.50%, p < 0.0001, F_(1,66)_ = 37.16, time x treatment: p = 0.0031, F_(10,66)_ = 3.047] (**Figure 8D**).

## Discussion

Mild traumatic brain injury (mTBI), or, more simply, concussion, is associated with several neuropsychiatric changes that are still neglected from a therapeutic management point of view. Safe pharmacological tools are needed for some of the concussion features related to the behavioral psychiatric changes such as aggressiveness, anxiety, obsessive compulsive like behaviors and depression, which are also reported in the DSM-V for post traumatic stress disorders (PTSDs). In the present study, we aimed at investigating whether pharmacological treatment with a new natural compound found in green and roasted coffee beans (Impellizzeri et al., 2016b), 2-pentadecyl-2-oxazoline (PEA-OXA), could be of any use for these comorbidities. Structurally related to palmitoylethanolamide (PEA), PEA-OXA shows both PEA-related and unrelated mechanisms of actions. Indeed, it has been demonstrated to produce anti-inflammatory effects mediated by inhibition of N-acylethanolamine hydrolyzing acid amidase (NAAA), the main enzyme responsible for the degradation of PEA, especially in white cell lineages such as macrophages and mast cells (Petrosino et al., 2017). Moreover, treatment with PEA-OXA normalized the otherwise reduced PEA and endocannabinoid levels found in the carrageenan-inflamed mouse paw, suggesting either a role of pro-drug for this molecule and/or indirect actions on the endocannabinoid system and its metabolic machinery (Petrosino et al., 2017). On the other hand, the anti-inflammatory effect of the drug was not abolished, as instead is observed for PEA, in PPARα null mice, suggesting additional molecular mechanisms at the basis of PEA-OXA pharmacological actions (Petrosino et al., 2017). In addition, alternative targets have also been proposed for this compound, involving the nuclear factor E_2_-related pathway (Cordaro et al., 2018). Thus, the multi-target nature of PEA-OXA makes it very interesting for chronic multi-factorial diseases, also in light of its good tolerability and safety profile (Impellizzeri et al., 2016b). In the present study we found differences in the pharmacological effect of PEA-OXA, compared to PEA, in a mouse model of mTBI that we have previously characterized in terms of behavioural phenotype using as a pharmacological tool the latter compound (PEA) (Guida et al., 2017). Indeed, while we found many similar pharmacological effects between the two molecules, PEA-OXA reduced the obsessive compulsive/repetitive-like behavior in the late phase of the disease, an endpoint that instead was not affected by PEA (**Supplementary Figure 1**).

We observed here another important difference between the pharmacological profile of PEA and PEA-OXA in terms of electrophysiological activity of the medial prefrontal cortex (*m*PFC) that represents a brain area highly involved in almost all the behavioral features of the concussion. In the previous study (Guida et al., 2017) we reported that PEA reduces the depressive-like behavior without any effect on the depressed neuronal activity 60 days after trauma. By contrast, in this study we found that the repeated administration with PEA-OXA reduced the depressive-like phenotype together with a clear effect at normalizing the neuronal activity in the *m*PFC, suggesting that this compound beneficially affects the impaired neuronal plasticity occurring in this brain area during the late consequences of mTBI injury.

We also demonstrated that mTBI mice showed memory retention impairment. Indeed, mTBI mice spent significant longer time to learn the position (quadrant) of the platform in the acquisition and reversal training and spent significant less time in the targeted quadrant in the probe test as compared to sham mice. Intriguingly, both sham and mTBI mice treated with PEA-OXA significantly spent less time to learn how to get to the targeted quadrant in the reversal training phase as compared to sham and mTBI mice treated with vehicle. Repeated PEA-OXA administration did not significantly prevent the memory impairment observed in the probe test, although it showed a trend in the amelioration of this task. Such a “nootropic” activity of the compound, together with the other beneficial effects, is intriguing and deserves further investigation, also in view of the possible mechanisms through which PEA-OXA may exert such effects.

In fact, due to its chemical structure, this compound might also act on other receptors such as norepinephrine α2 receptors. Therefore, we attempted to identify the possible activity of PEA-OXA on α2 receptors. We used a cAMP assay in HEK-293 cells overexpressing the α2 receptors. Our results demonstrate that PEA-OXA behaves as a α2 antagonist. Accordingly, we performed a binding assay for the α2 receptor and found that the compound shows indeed a high affinity for the receptor (see **Supplementary Material**). In order to verify the α2-mediated pharmacological effect, we performed open field task by microinjecting into the mPFC the selective α2 agonist dexomedetomidine, alone or in combination with PEA-OXA. Interestingly, we found a reduced locomotion induced by dexomedetomidine, which was prevented by the co-injection with PEA-OXA, confirming a possible α2-mediated mechanism. However, PEA-OXA microinjection alone was associated with increased locomotory activity, therefore we measured the dopamine levels after PEA-OXA microinjection. As suspectable PEA-OXA, increased the levels of Dopamine in the mPFC. This neurobiochemical effect deserves further investigation for a potential role of this secondary plant metabolite in neuropsychiatric disorders associated with dopamine changes.

The present new insights in the mechanism of action of PEA-OXA are potentially relevant to its pharmacological effects *in vivo*. In fact, it is known that several very effective antipsychotic drugs (i.e. clozapine) also show a similar pharmacodynamic profile (Marcus et al., 2010). Also other drugs in psychiatry act on those receptors such as mirtazapine and mianserine (De Boer et al., 1996).

Moreover, α2 blockade has been demonstrated to increase dopamine levels in cortical areas, thereby reducing the worsening effect of classical (also known as “typical”) antipsychotic drugs (Uys et al., 2017). We also showed here that the compound administered at the dose of 20 mg/kg per os significantly increased GABA levels in the *m*PFC, without evident effects on glutamate release. This effect is likely to be important in psychosis, in which GABAergic interneurons play a key role in maintaining homeostasis of neural circuitries (in dopamine or serotonin producing neurons) (Kolata et al., 2018). Indeed, it has been recently demonstrated in patients with schizophrenia and depression, that the negative symptoms are associated to GABA level reduction in the *m*PFC and hippocampus (Kolata et al., 2018). The increased levels of GABA by PEA-OXA could be mediated by blockade of the α2 receptor, which is massively expressed in GABAergic interneurons (Manns et al., 2003). Finally, the catecholaminergic-mediated mechanisms could also result in an anti-neuro-inflammatory effect that is important in the early phase of mTBI and other psychiatric diseases, in which neuroinflammation is being increasingly suggested to play a key role (Schimmel et al., 2017). Intriguingly, it has been also suggested that, although the stimulation of the α2 noradrenergic receptors reduces the proliferation of T-lymphocytes, its blockade, together with a block of the I_2_ imidazoline receptors, reduces nitric oxide (NO), interferon γ (IFN-γ) and interleukin 2 (IL-2) release from splenocytes (Priyanka and Thyagarajan, 2013), suggesting an overall anti-inflammatory effect of α2 antagonists.

In summary, these data pave the way to the clinical investigation of the possible treatment of post-TBI-associated behavioral disorders, especially depressive-like behaviors, with PEA-OXA. Moreover, the pharmacodynamic profile of the drug is interesting since it shares several targets with PEA, but the efficacy and spectrum of its effects are strengthened and widened by its capability to modulate other receptors. Further studies are needed to better understand the pharmacodynamics of PEA-OXA and its possible use in other psychiatric diseases such as mood disorders, depression, and schizophrenia.

## Data Availability Statement

All datasets generated for this study are included in the article/**Supplementary Material**.

## Ethics Statement

The animal study was reviewed and approved by the Ethical Committee of the University of Campania “L. Vanvitelli” and the University of Naples “Federico II, Naples.”

## Author Contributions

SB, MI, CC, FI, CB, RI, and FR performed experiments. FB, FG, LL, and SB analyzed the data and planned experiments. LL, VD, AC, MG, and SM designed the study, wrote, and sponsored the paper.

## Funding

The paper is supported by PRIN 2015 of SM and LL. MI and SB are supported by Epitech group.

## Conflict of Interest

The authors declare that the research was conducted in the absence of any commercial or financial relationships that could be construed as a potential conflict of interest.

The reviewer, DDG, declared a past co-authorship with several of the authors, MI, FI, FG, VD, SM, LL, to the handling editor.
