# Supplementary material for: Treatment With 2-Pentadecyl-2-Oxazoline Restores Mild Traumatic Brain Injury-Induced Sensorial and Neuropsychiatric Dysfunctions
Source: Front Pharmacol. 2020 Feb 25;11:91. doi: 10.3389/fphar.2020.00091 (PMC7052365; doi:10.3389/fphar.2020.00091)
Supplement: Supplementary file 1 [file DataSheet_1.docx]

**Supplementary results**

**PEA did not change mTBI-Induced compulsive behaviour**

At day 60 post trauma, the treatment with PEA (10 mg/kg, i.p.) did not reduce the number of buried marble and digging events in mTBI mice (13.8 ± 1.3 and 412.0 ± 11.6, respectively) as compared to mTBI/vehicle mice (11.8 ± 0.4 and 363.2 ± 35.9, respectively), differently from PEA-OXA that significantly reduced this compulsive behaviour (Fig. 1supp). Instead, no differences were observed between sham mice treated with PEA (6.3 ± 2.3 and 212.6 ± 92.7 respectively) and with PEA-OXA (4.6 ± 1.5 and 95.5 ± 17.4 respectively) as compared to sham mice treated with vehicle in both parameters (4.1 ± 0.9 and 84.5 ± 13.9 respectively) (Fig. 1supp).

**Supplementary Figure legend**

**Fig. 1supp Effects of repeated administration (14 days) of vehicle (Pluronic acid 5%), PEA (10 mg/Kg, i.p.) or PEA-OXA (10 mg/kg, o.s.) on compulsive behaviour in sham and mTBI mice.** (A and B) Number of buried marbles and digging events in marble burying test, respectively. Data are represented as mean ± SEM of 8 mice per group. * indicate significant differences compared to sham/vehicle, ^°^ indicate significant differences compared to mTBI/vehicle and ^§^ indicate significant differences compared to mTBI/PEA. *P* < 0.05 was considered statistically significant. One-way ANOVA, followed by Bonferroni post hoc test was performed.
