# Supplementary figures and images for: Treatment With 2-Pentadecyl-2-Oxazoline Restores Mild Traumatic Brain Injury-Induced Sensorial and Neuropsychiatric Dysfunctions
Source: Front Pharmacol. 2020 Feb 25;11:91. doi: 10.3389/fphar.2020.00091 (PMC7052365; doi:10.3389/fphar.2020.00091)

## Slide 1
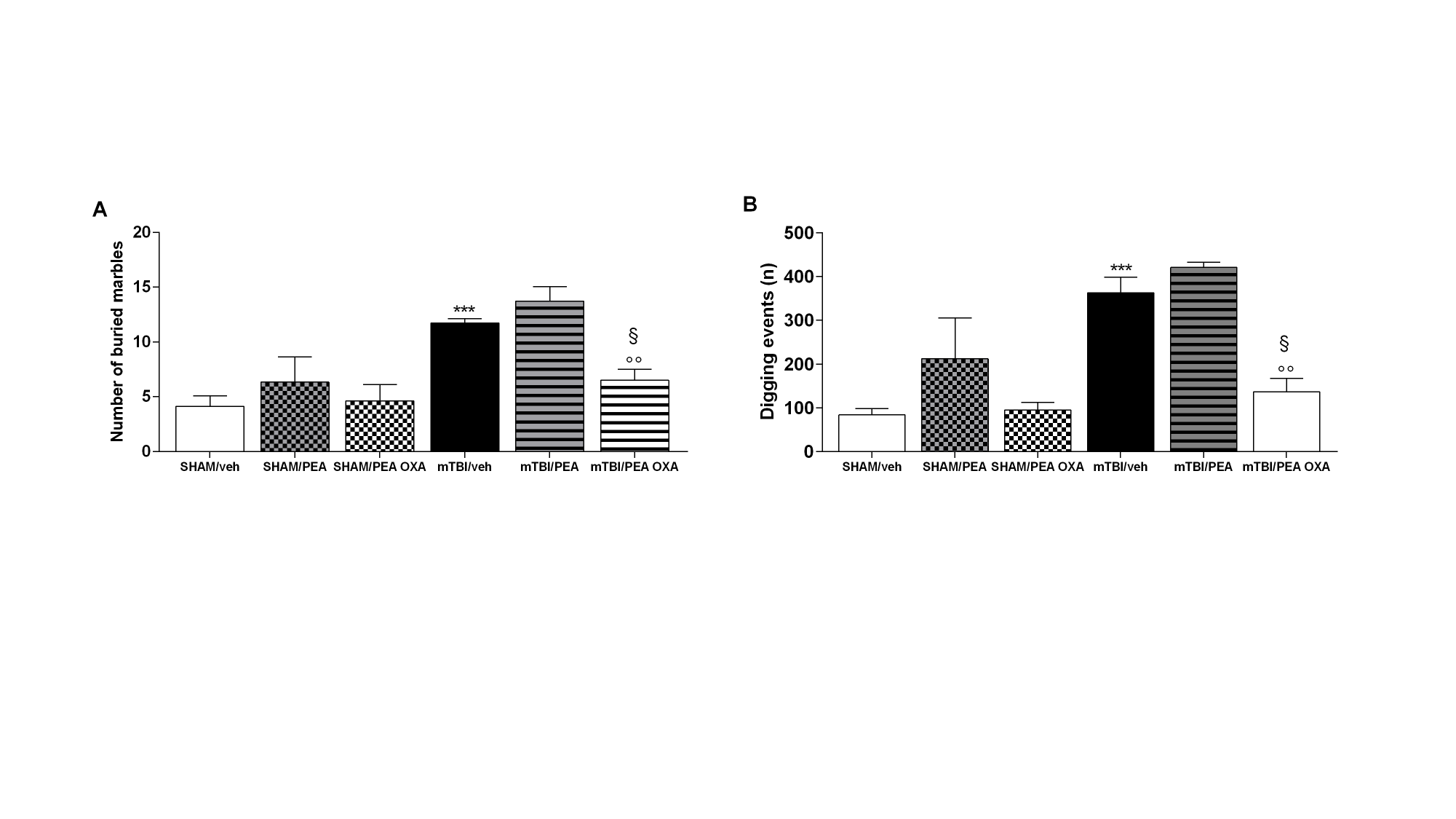

Supplement: Supplementary file 2 [file Presentation_1.pptx]
