# Supplementary material for: Treatment With 2-Pentadecyl-2-Oxazoline Restores Mild Traumatic Brain Injury-Induced Sensorial and Neuropsychiatric Dysfunctions
Source: Front Pharmacol. 2020 Feb 25;11:91. doi: 10.3389/fphar.2020.00091 (PMC7052365; doi:10.3389/fphar.2020.00091)
Supplement: Supplementary file 3 [file Table_1.doc]

**SUPPLEMENTARY MATERIAL**

**Radioligand binding assays at α2 adrenergic receptors**

The α2-adrenergic profile of PEA-OXA was evaluated by radioligand competition binding assays using the selective antagonist [^3^H]MK912 to label α2-adrenergic receptors, similarly to previously described procedures.^S1^ The affinity value, expressed as IC_50_, is reported in Table S1 together with that of MK912, included as reference compound.

**Table 1.** Binding affinity values, expressed as IC_50_ (μM), of PEA-OXA and MK912.

| Compound | IC_50_ (µM) |
| --- | --- |
| PEA-OXA | 0.75±0.06 |
| MK912 | 0.0012±0.002 |

Competition curves were analyzed by non linear curve fitting (GraphPad, version 7 for Windows).

***Preparation of cerebral cortical membranes.***

Cortical membranes were prepared similarly to previously described procedure.^S1^ Male Wistar rats (Charles River, Calco-BG Italy) were sacrificed by decapitation, their brains were rapidly extracted and the cerebral cortex dissected on a cold Petri dish. The tissue was homogenized in 25 volumes of ice cold Tris buffer (50mM Tris HCl, 5 mM EDTA: pH 7.4 at 25 °C). The homogenate was then centrifuged at 50,000 g for 15 min at 4 °C. After a second wash, the pellet obtained was resuspended in Tris buffer (50 mm Tris HCl, 0.5 mm EDTA; pH 7.4 at 25 °C) before undergoing two further washes. The final pellet was resuspended in assay buffer for direct use in binding assays or stored under liquid nitrogen.

***Binding assay***

α2-Adrenergic competition binding assays were performed by incubating 100 μl of membranes suspension (75-100 µg protein/sample) in triplicate with 0.8 nM [^3^H]MK912 (PerkinElmer Life Sciences) in the presence or absence of various concentration of test compounds (25 µl), in a final volume of 0.25 ml Tris assay buffer (50mM Tris HCl, 0.5 mm EDTA; pH 7.4 at 25 °C). Non-specific binding was determined in the presence of 1 μM MK912 (Sigma). All solution prepared with radiolabelled or cold MK912 were performed in polypropylene microplate or tubes. The incubation, performed in polypropylene microplates, started adding the membranes and was carried out for 60 min at 25°C. The bound radioligand was separated by rapid filtration on Unifilter glass‑fiber GF/B filter microplate. Filtrates were washed four times with ice- cold assay buffer; filters were dried and for 30 minutes at 30 °C and then 0.05 ml of MICROSCINT-20 (Packard) were added. Plates were counted after at least 1 hour of stabilization.

***References***

S1. Brown. C. M.; McKinnoc, A. C.; McGrath, J. C.; Spedding, M.; Kilpatrik, A. T.,

α_2_-Adrenoceptor subtypes and imidazoline-like binding sites in rat brain. *Eur J Pharmacol* **1990**, *99,* 803-9*.*
